# Carbon Ion Radiotherapy at the Gunma University Heavy Ion Medical Center: New Facility Set-up

**DOI:** 10.3390/cancers3044046

**Published:** 2011-10-26

**Authors:** Tatsuya Ohno, Tatsuaki Kanai, Satoru Yamada, Ken Yusa, Mutsumi Tashiro, Hirofumi Shimada, Kota Torikai, Yukari Yoshida, Yoko Kitada, Hiroyuki Katoh, Takayoshi Ishii, Takashi Nakano

**Affiliations:** Gunma University Heavy Ion Medical Center, Gunma University, Showa 3-39-22, Maebashi, Gunma 371-8511, Japan; E-Mails: kanai@showa.gunma-u.ac.jp (T.K.); satoru@showa.gunma-u.ac.jp (S.Y.); yusa@gunma-u.ac.jp (K.Y.); tashiro@gunma-u.ac.jp (M.T.); shimada@gunma-u.ac.jp (H.S.); torikai@gunma-u.ac.jp (K.T.); yyukari@gunma-u.ac.jp (Y.Y.); yokon@gunma-u.ac.jp (Y.K.); hkatoh@gunma-u.ac.jp (H.K.); t-ishii@showa.gunma-u.ac.jp (T.I.); tnakano@med.gunma-u.ac.jp (T.N.)

**Keywords:** carbon ion radiotherapy, cancer treatment, high LET, facility set-up

## Abstract

Carbon ion radiotherapy (C-ion RT) offers superior dose conformity in the treatment of deep-seated tumors compared with conventional X-ray therapy. In addition, carbon ion beams have a higher relative biological effectiveness compared with protons or X-ray beams. C-ion RT for the first patient at Gunma University Heavy Ion Medical Center (GHMC) was initiated in March of 2010. The major specifications of the facility were determined based on the experience of clinical treatments at the National Institute of Radiological Sciences (NIRS), with the size and cost being reduced to one-third of those at NIRS. The currently indicated sites of cancer treatment at GHMC are lung, prostate, head and neck, liver, rectum, bone and soft tissue. Between March 2010 and July 2011, a total of 177 patients were treated at GHMC although a total of 100 patients was the design specification during the period in considering the optimal machine performance. In the present article, we introduce the facility set-up of GHMC, including the facility design, treatment planning systems, and clinical preparations.

## 1. Introduction

The application of radiotherapy (RT) is based on the fundamental principle of achieving precise dose localization in the target lesion while causing minimal damage to surrounding normal tissues. Charged particle therapy with protons and carbon ions allows highly localized deposition of energy that can be utilized for increasing radiation doses to tumors while minimizing irradiation to adjacent normal tissues. Additionally, carbon ion beams have various biological advantages in terms of high linear energy transfer, including a decreased oxygen enhancement ratio, a diminished capacity for sublethal and potentially lethal damage repairs, and reduced cell cycle-dependent radiosensitivity compared with those observed with protons or X-ray beams [1].

In 1946, Wilson hypothesized that the unique property of charged particles can be used in cancer treatment. Lawrence and Tobias at the Lawrence Berkeley Laboratory first initiated the medical application of proton beams between 1954 and 1957. Since then, more than 84,000 patients have been treated with charged particle therapy in the world [2]. Although there are 30 proton therapy centers and five carbon ion radiotherapy (C-ion RT) centers in operation, half of them began charged particle therapy with protons or carbon ions just during the last decade. At present, the construction of more than 15 hospital-based facilities is planned within the next 10 years. In particular, most of them will be based not in physics institutes but in hospitals with long-standing experience in modern photon RT.

This expansionary trend of charged particle therapy has attracted growing interest in setting up such a facility, although the costs are high and much time is required compared with conventional photon therapy. The National Institute of Radiological Sciences (NIRS) put into operation the first C-ion RT facility in Japan using the Heavy Ion Medical Accelerator in Chiba (HIMAC), and it has been used to treat cancers of various types in more than 6,000 patients [3]. Although many promising clinical outcomes have been reported from NIRS [4,5], the high construction and operation costs of the accelerator system was one of the problems facing subsequent facilities being planned for C-ion RT. Based on the design and R&D studies carried out by NIRS, the size and cost of the machine could be reduced to one-third of those of NIRS. By using a compact prototype of the accelerator system, C-ion RT for the first patient at the Gunma University Heavy Ion Medical Center (GHMC) took place on schedule in March 2010. In the present article, we introduce the facility set-up of GHMC, including the facility design, treatment planning systems, and clinical preparations.

## 2. Facility Design

### 2.1. Building Design

The C-ion RT facility is located at Gunma University Hospital. The basic and execution designs of the building were started in April and July 2006, respectively. The construction started in February 2007 and was completed in October 2008. The building has two stories above ground and one below, and uses reinforced concrete and a partial steel frame with a construction area of 3,140 m^2^ and a total floor area of 6,280 m^2^. The size of the facility is about 65 m × 45 m, approximately 1/3 of HIMAC. The height is ∼20 m at maximum for the vertical beam course. The building structure is placed directly on a gravel stratum lying about 4 m below ground; therefore, the floor level (B1) is 2 m below ground level. The excavated soil was partially used for banking around the building, contributing to decreasing the height of the building and hopefully ameliorating feelings of unease from living in the “shadows of a giant” for surrounding residences. Of course, it is also advantageous for radiation shielding, as the thickness of the concrete wall could be reduced, and thereby the cost of the building as well.

**Figure 1.**
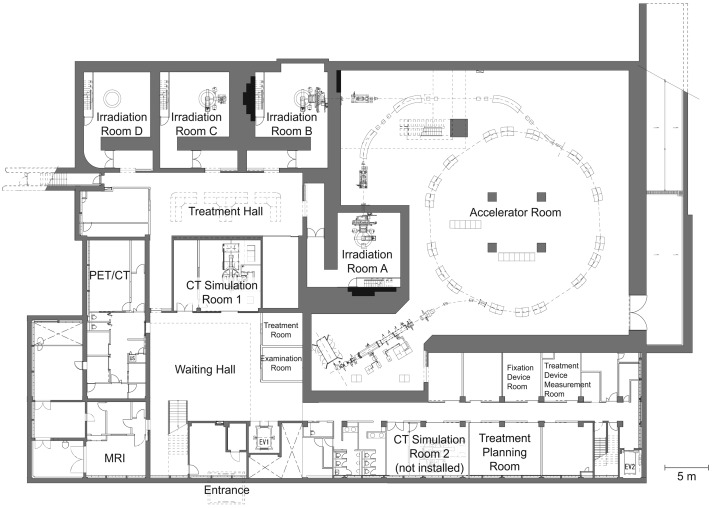
Layout of the B1 floor.

The layout of the bottom floor is shown in Figure 1. The facility contains three treatment rooms with four irradiation ports (horizontal; Room A, horizontal + vertical; Room B, and vertical; Room C) and a room with a vertical port intended for R&D of beam delivery system and biology experiments. In addition, dedicated CT simulators, PET/CT, and MRI were installed to support efficient and accurate treatment procedures. An examination room and a treatment room used for pre- and post-irradiation procedures in addition to those for the above mentioned modalities are arranged around a waiting hall for patients and staff accessibility.

**Figure 2.**
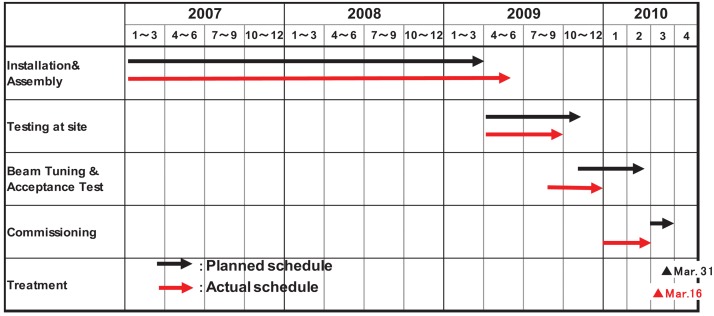
Planned and actual construction schedule of the treatment system at GHMC.

Figure 2 shows the planned and actual schedules of the treatment system at GHMC. It took about three years from the start of construction to treatment of the first patient. Intensive weekly meetings were carried out between GHMC and vendor (Mitsubishi Electric Corporation) or the administration of Gunma University. In addition, experts from NIRS joined the meetings if necessary. At the beginning phase of the installation, the schedule was behind due to delays in the accelerator production, thus, parallel works were rescheduled in order to recoup the delays. Beam testing was started on 12 August, and acceleration up to 400 MeV/n was attained on 25 August.

### 2.2. Ion Source and Linear Accelerator

**Figure 3.**
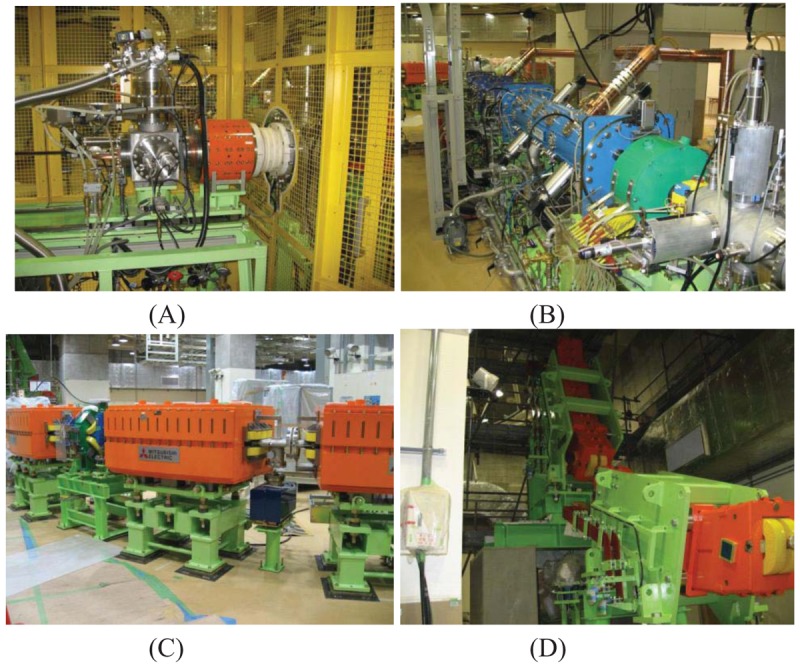
(**A**) 10 GHz carbon ion source; (**B**) RFQ linac and APF linac; (**C**) GHMC synchrotron ring; (**D**) High energy beam transfer line (vertical bending).

A compact electron-cyclotron resonance (ECR) ion source is installed in this facility (Figure 3A). Permanent magnets are assembled to generate both of a sextupole and a mirror magnetic field. Compared to the conventional electro-magnetic type ECR ion source, the permanent magnet unit is effective for size reduction, easy operation and simple maintenance. A microwave amplifier for the ECR is equipped with a traveling wave tube, and the operating frequency is 10 GHz. Non-radioactive methane gas is used for the ion generation. Four+ carbon ions are initially extracted from the ECR ion source with energy of 10 keV/n. The extracted beam current is approximately 200 μA. A low energy beam transfer line is composed of an Einzel lens, a 90-degree bending magnet, a vertical and horizontal beam slit, a beam attenuator and electro-static triplet focusing lens, and a solenoid magnet. This ECR ion source is designed to start operating without requiring any warm-up for the C-ion RT.

Two succeeding linear accelerators are radio-frequency quadrupole linac (RFQ linac) and alternating phase focusing linac (APF linac) (Figure 3B). The RFQ linac accelerates the carbon ion beam up to 600 keV/n, and the APF linac accelerates the carbon beam up to 4 MeV/n. Both linacs operate at a frequency of 200 MHz. The length of the APF linac is only 3.67 m, thereby greatly contributing to the compactness of the injector system.

### 2.3. Synchrotron and Beam Transfer Line

The accelerator complex is composed of a synchrotron and a high energy beam transfer line (Figures 3C,D). The circumference of the heavy ion synchrotron is 63.3 m. Eighteen bending magnets and six pairs of focusing/defocusing magnets are aligned to generate an operation tune of (νx/νy = 1.68/1.23), and an extraction tune of 5/3, including injection/extraction electric septum magnets (ESI/ESD) and two pairs of sextupole magnets for 3rd resonance extraction. Maximum carbon beam energy is 400 MeV/n. Each cycle of excitation pattern of the synchrotron is normally 2.7 seconds. Injection time is 0.1 seconds, acceleration and deceleration are 0.7 seconds, and 1.2 seconds is for the extraction. The extracted beam has a maximum intensity of 1 × 10^9^ particles/ seconds, which is equivalent to 5 GyE/min. The extracted beam emittance is designed as εx/εy = 2.4/5.0 πmm-mrad, with a momentum spread of 0.1%. The distance between the final bending magnet and the isocenter of all treatment ports is designed for 9 m to acquire treatment room interchangeability, in preparation for machine failure or maintenance.

### 2.4. Beam Delivery System

The beam delivery system adapts the high-energy carbon beams generated by the accelerator complex to therapeutic usage and delivers them to the patient lying in the treatment room. The facility has three treatment rooms with four irradiation courses, each of which possesses its own beam delivery system. Each beam delivery system has the same layout and functions to ensure uniformity in patient treatments and ease of maintenance.

**Figure 4.**
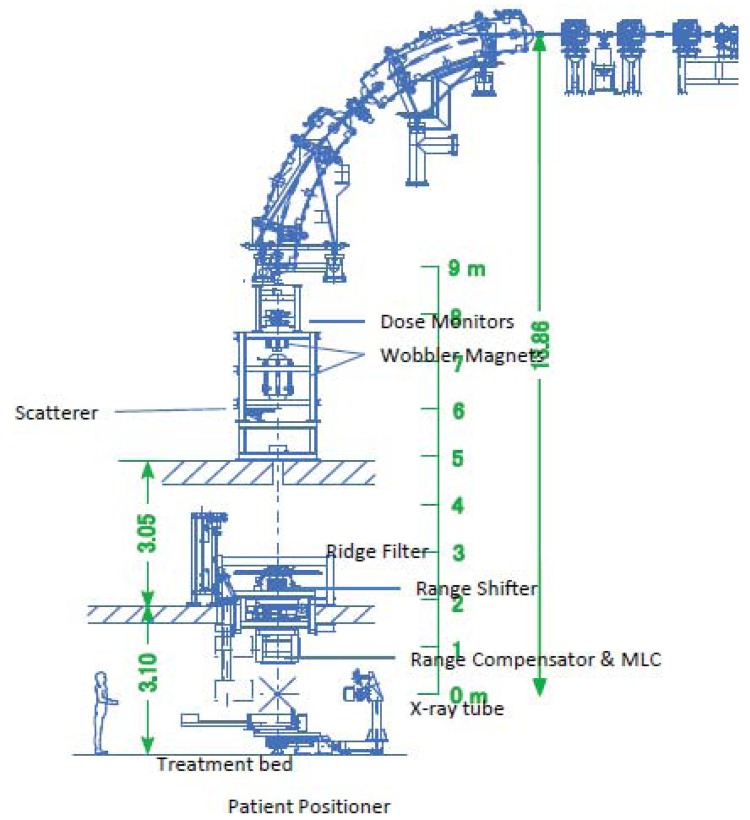
Beam delivery system.

A beam delivery system comprises several devices, *i.e.*, dose monitors, wobbler magnets, a scatterer, a ridge filter, a range shifter set, a multi-leaf collimator (MLC), a compensator and a patient positioner system, for precisely aiming the therapeutic beams at the planned target volume (PTV) by adopting a beam wobbling method. A schematic view of a beam delivery system is illustrated in Figure 4.

The basic specifications of the beam delivery system are summarized in Table 1. The required maximum residual range should be over 25 cm in water, which is sufficiently large to reach tumors located deeply in the patient's body. The maximum irradiation field size is 15 cm × 15 cm under the MLC fully-opened condition. A typical dose rate required for treatment is about 5 GyE/min, and several minutes of irradiation can fulfill the prescribed dose. Inhomogeneity of the irradiation field is required to be within ±2.5%.

Irradiation courses with short geometry require a downsized scale of the entire facility. The length of the irradiation courses at this facility is 9 m, whereas the existing facility, HIMAC at NIRS in Japan, has 11-m long irradiation courses.

Employing the conventional single circle wobbling method in the shortened irradiation course requires a thick scatterer to broaden the beam to create a large irradiation field. The beam's energy loss in the chosen thick scatterer can cause a shortage of the residual range in the patient's body. Introducing a spiral wobbling method in which a thin scatterer can be used meets requirements for both a long residual range and a large irradiation field size. Details for these wobbling methods can be found in reports [6] and [7].

### 2.5. Irradiation Methods

Respiratory displacement causes an unintended dose distribution to the target and the tissue surrounding the target during irradiation. The use of a respiration-gated irradiation system (Anzai Respiratory Gated System, AZ-733V), which is fully implemented in the treatment control system, enables a reduction in these types of irradiation errors. The conventional irradiation method, in principle, allows a part of the SOBP volume to protrude from the target into normal tissue on the proximal side. The layer stacking conformal irradiation method can reduce this unwanted extra-dose volume in normal tissue through dynamic changes in the MLC aperture during stacking irradiation [8,9]. One of the three treatment rooms has two irradiation courses, *i.e.*, vertical course and horizontal course. This configuration allows the patient to be irradiated from two directions in a row with the same patient positioning, thus shortening the treatment time.

## 3. Treatment Planning System (TPS)

At GHMC, we introduced a treatment planning system (Xio-N) newly developed for the compact facility of carbon therapy. Xio (Elekta) has been used as a platform of the Xio-N system. K2DOSE has been added to the Xio platform to calculate dose distributions by carbon beams in patients. The dose calculation engine (K2DOSE) communicates with the platform in order to display the dose distributions seamlessly at the Xio platform. We set up the DICOM server for ion therapy, which we call the DICOM-ION server. The DICOM-ION server can store the calculated TPS data and communicate with other modalities in DICOM-RT format, such as RT ION Plan, RT Structure Set, RT Dose, and CT Image.

K2DOSE adapted the pencil beam method to calculate dose distributions in patients for carbon and also proton therapy with the broad beam method. In carbon therapy, two kinds of dose distributions, clinical and physical, are used in TPS. The physical dose denotes the absorbed dose and clinical dose includes the RBE. The clinical dose distributions are calculated according to the NIRS method [10], which is based on the LQ model of survival curves for human salivary grand tumor cell (HSG). Physical dose and LET distributions are calculated using the pencil beam algorithm for the carbon beam. From the LET distributions, α and β are obtained. The clinical dose is calculated from the physical dose and the calculated αand β in the LQ model. TPS can calculate the dose distributions by various irradiation methods, a layer-stacking irradiation method, a spiral wobbling method, and the single wobbling method.

## 4. Clinical Preparations

### 4.1. Staff

GHMC in connection with Gunma University Hospital offers all modern radiation techniques including stereotactic radiotherapy, intensity-modulated radiotherapy, image-guided external beam radiotherapy, and image-guided brachytherapy. The Department of Radiation Oncology of Gunma University Hospital has a close and interwoven relationship with GHMC. The dedicated staff for C-ion RT at GHMC consists of five radiation oncologists, six physicists, two treatment planning assistants, eight operation staff members, three oncology nurses, and six radiology technologists. About half of the staff was trained in C-ion RT at NIRS for between 3 months and 2 years. The remaining staff undertook on-the-job training at GHMC. Further staff employment is planned via on-the-job training at GHMC as the number of patients increases.

### 4.2. Clinical Indications

Many phase I/II dose escalation and phase II studies for various tumor sites have been carried out at NIRS since 1994 [5]. Although promising clinical outcomes have been reported from NIRS, it is of interest whether the efficacy of carbon ion radiotherapy from a single institution can be reproduced in other facilities when optimal doses and fractionations are used for a similar patient population. At GHMC, the efficacy and safety of carbon ion radiotherapy were reviewed for each tumor type, and then the best available dose and fractionation schedules determined at NIRS were adopted for our clinical protocols. All clinical protocols have been prepared by the disease-specific committees consisting of radiation oncologist, surgical oncologist, medical oncologist, and pathological oncologist. The protocols were reviewed by the Internal Review Board of Gunma University Hospital. Between March 2010 and July 2011, a total of 177 patients were treated at GHMC. The most common site of cancer was prostate (n = 139), followed by lung (n = 14), liver (n = 9), bone and soft tissue sarcoma (n = 8), and head and neck tumor (n = 7).

### 4.3. Estimated Number of Patients

An estimation model of the need for particle radiotherapy was constructed as follows. First, the region consisting of Gunma prefecture and four adjacent prefectures (Tochigi, Niigata, Nagano, Saitama) was specified. In 2003, the total population of this region was 15,748,026 (12.3% of the population of Japan). Based on the Japanese cancer registration, the estimated number of cancer patients of this region was 79,172 in 2003 [11]. In this region, only GHMC offers particle beam RT.

Second, the total number of patients was calculated for head and neck, rectum, liver, lung, prostate, and bone and soft tissue by using data on cancer incidence statistics and the size of the regional population [11]. Third, certain proportions of the clinical attributes (stage, tumor size, histology, *etc.*) of each cancer site were determined according to regularly structured surveys by the Cancer Society of Japan, cancer registration, published manuscripts, and textbooks [12]. Regarding this process, Japanese data from the period of 1999 to 2003 were applied as much as possible. The potential number of patients in the region who might benefit from C-ion RT at GHMC was calculated for each site. C-ion RT was potentially indicated for 8,085 patients per year and realistically for 1,527 patients per year, corresponding to 10% and 2% of the newly diagnosed cancer patients in the region. Prostate cancer (541 patients) followed by lung cancer (436 patients), and liver cancer (313 patients) were the most commonly diagnosed cancers.

### 4.4. Cost Effectiveness

We evaluated the cost-effectiveness of carbon ion radiotherapy compared with conventional multimodality therapy in the treatment of patients with locally recurrent rectal cancer [13]. Direct costs for diagnosis, recurrent treatment, follow-up, visits, supportive therapy, complications, and admission were computed for each individual using a sample of 25 patients presenting with local recurrent rectal cancer at NIRS and Gunma University Hospital. Patients received only radical surgery for primary rectal adenocarcinoma and had isolated unresectable pelvic recurrence. Fourteen and 11 patients receiving treatment for local recurrence between 2003 and 2005 were followed retrospectively at NIRS and Gunma University Hospital, respectively. Treatment was carried out with C-ion RT alone at NIRS, while multimodality therapy including three-dimensional conformal radiotherapy, chemotherapy, and hyperthermia was performed at Gunma University Hospital. The 2-year overall survival rate was 85% and 55% for C-ion RT and multimodality treatment, respectively. The mean cost was 4,803,946 JPY for the C-ion RT group and 4,611,100 JPY for the multimodality treatment group. The incremental cost-effectiveness ratio for C-ion RT was 6,428 JPY per 1% increase in survival. The median duration of total hospitalization was 37 days for C-ion RT and 66 days for the multimodality treatment group.

## 5. Set-up for First Treatment

### 5.1. Biophysical QA

**Figure 5.**
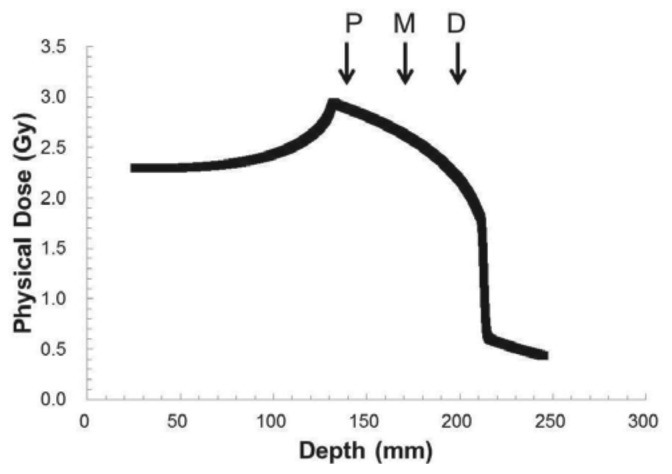
Physical depth-dose distribution of carbon ion beams. Depth-dose distribution of carbon ion beams with 80 mm spread-out Bragg peak (SOBP). Arrows indicate three positions of cells irradiated with carbon ions. Human salivary gland tumor cells were irradiated at position P (30 mm upstream), D (30 mm downstream) of middle position (M).

Biological study was performed in homogeneous and inhomogeneous materials to answer the following questions: is the biological SOBP dose the predicted distribution at GHMC? Is the treatment planning system (TPS) validated at GHMC? Figure 5 shows the physical depth-dose distribution of carbon ion beams used in the present study.

**Figure 6.**
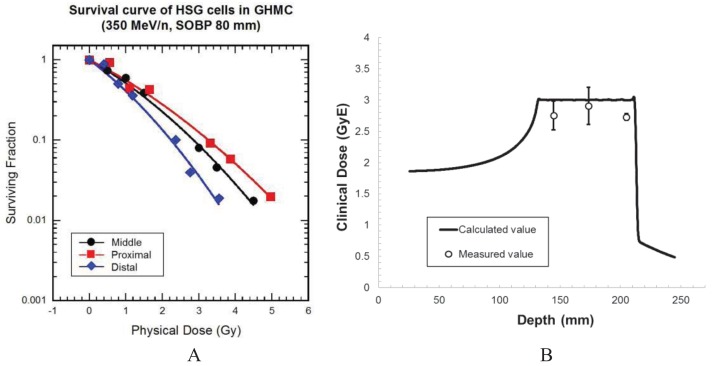
(**A**) Human salivary gland tumor cell survivals after irradiation with carbon ions. Data are obtained by irradiating cells at positions shown in Figure 4 as proximal (P), middle (M), distal (D), respectively; (**B**) The comparison between the measured values and the calculated values.

Cultured cells from human salivary gland tumor (HSG cells) were irradiated at three points. A position labeled P (proximal) was 30 mm upstream of the center M (middle) of 80 mm SOBP, whereas position D (distal) was 30 mm downstream of the center M. X-ray (200 kV) was used as a reference. Relative biological effectiveness (RBE) values at each point were calculated from survival curves. RBE values were calculated from cell survival curves at a dose that would reduce cell survival to 10% (D10). The D10 values were obtained from the α and β parameters for each survival data when survival curves were drawn using the linear-quadratic model. Figure 6A shows the cell survival curves of 350 MeV/n carbon ion SOBP beams. The slope of the cell survival curves became steeper as the depth in SOBP increased. The RBE values at the D10 dose level were 1.75 at P, 2.01 at M, and 2.53 at D in SOBP. When the cell survival values were compared with calculated values by the NIRS method [10], the value of measured survival data at M was 3% lower than the calculated one (Figure 6B).

**Figure 7.**
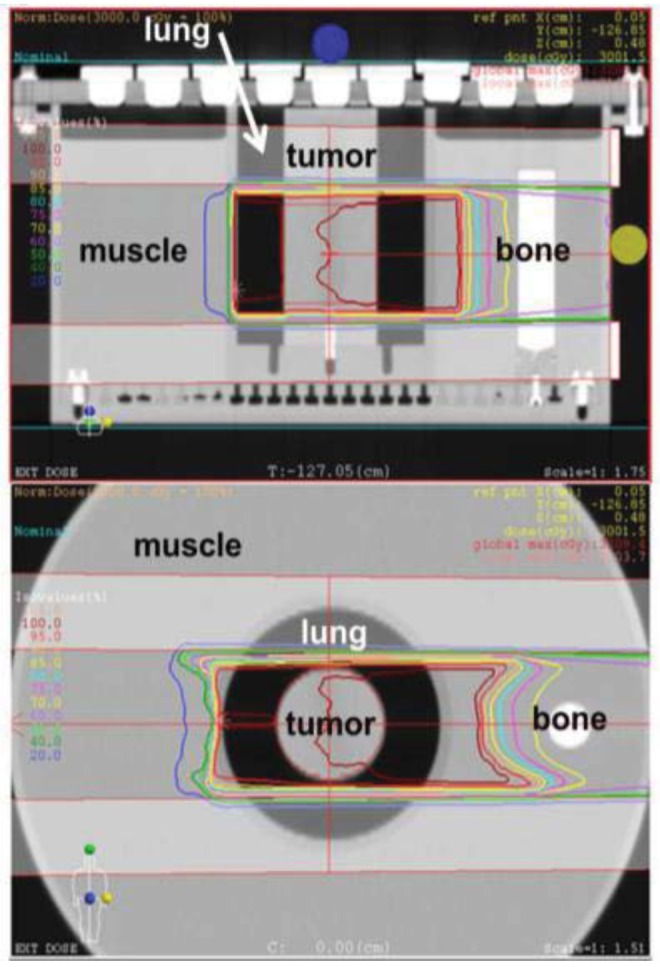
Dose distribution in inhomogeneous phantom including equivalent materials such as lung, bone and fat.

For QA/QC of TPS, accuracy of TPS was evaluated biologically in the inhomogeneous system. HSG cells were enclosed in a cell tube and embedded within an inhomogeneous phantom including equivalent materials such as lung, bone and fat (Figure 7).

The cell survival values of carbon ions were measured by colony formation of HSG cells, and were compared with the predicted values by the new treatment planning system Xio-N. The values of the measured data at the center of the tumor were 16% lower than the predicted ones. The values of the measured physical dose using the Farmer chamber were 1% lower than the calculated physical dose by Xio-N. These results suggest that the difference in survival data between homogeneous and inhomogeneous systems was about 10%. Not only the dose but also the radiation quality affects the biological responses in carbon ion therapy. The difference of biological effectiveness in the inhomogeneous system may be caused by radiation quality. This should be checked through detailed analysis of the radiation quality using Monte Carlo calculation and radiation quality measurements. Further investigation using the inhomogeneous phantom and various cells, as well as biological and physical studies will be promoted for the treatment of various organs.

### 5.2. Clinical Flow

**Figure 8.**
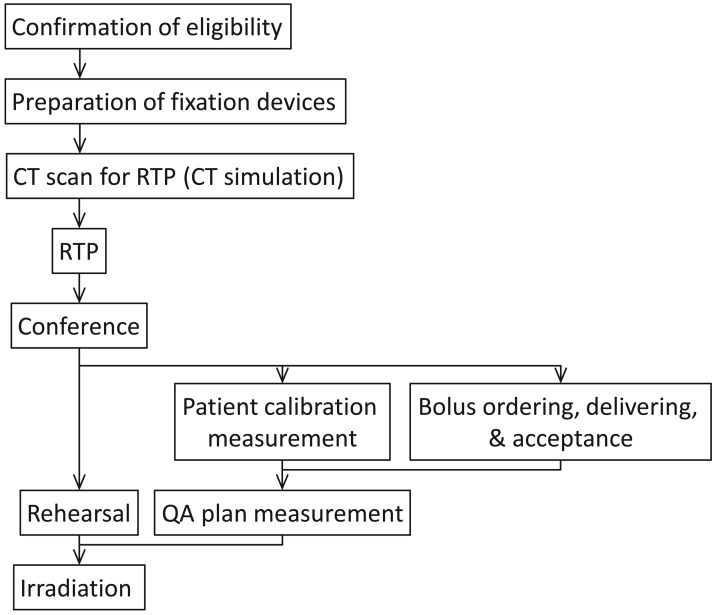
Typical clinical flow. RTP: radiation treatment planning.

The typical clinical flow is schematically shown in Figure 8. After confirmation of eligibility for carbon ion therapy, written informed consent was obtained from all patients. The first preparatory step is the fabrication of fixation devices for each patient. As fixation devices, MOLDCARE ^®^ cushions, composed of soft fabric bags containing expanded polystyrene beads coated with a moisture-cured polyurethane resin, and thermo-plastic shells of 3-mm thickness were used. Since only fixed beam ports are available, the patient couch can be rotated if necessary. The thickness of the shell is greater than conventional ones. The most common treatment position is the supine position with vertical or horizontal beam, but a fixation device for the prone position is also available when the posterior beam is used.

In the treatment position with the fixation devices, CT scan was carried out using Toshiba Aquilion LB with scan conditions of 1-second scan speed, 2-mm slice thickness and 550-mm FOV. Reconstructed CT images were used for treatment planning. For prostate cancer the treatment plan was made with three beam directions (vertical, left side, and right side) and a clinical dose of 57.6 GyE/16 fractions. After the treatment plan was approved at a staff conference, bolus range compensators made of polyethylene were ordered to be fabricated, and they were designed to adjust the beam ranges to the shapes of distal edges of the target for all beam ports. The shape of the delivered bolus was verified for acceptance using a coordinate measuring machine.

The plan data were sent to the beam delivering system. In order to obtain patient calibration constants, *i.e.*, relations between planned and delivered doses, patient-reference-depth measurements were carried out for all beams using a water phantom and an ionization chamber. Furthermore, as physical QA, planned physical doses were verified by comparing them with measured dose profiles at some selected points using a “QA plan”, in which the original plan was converted to a water phantom with the same beam settings.

Before irradiation, a rehearsal was carried out for the patient in the irradiation room or the CT simulation room. At the rehearsal, patient registration was done using the positioning system between digital reconstructed radiograph (DRR) derived from the planning CT data and X-ray fluoroscopic images from the X-TV system using a Shimadzu flat-panel detector (FPD). The obtained X-ray images were used for patient positioning at daily irradiations.

Instead of the rehearsal, X-ray fluoroscopic images can be obtained at the same time as the CT scan at the CT simulation room. In this case, just after the CT scan, the CT images are sent to a temporary treatment planning system (customized Xio-N) equipped in the room. Then, after the iso-center and beam ports are set, corresponding DRR images are sent to the positioning system. The patient couch is moved from the CT position to the treatment simulation position. Then X-ray images can be obtained through patient positioning. This process enables us to omit the rehearsal from the patient clinical flow, leading to saving patients' steps, and this is called ‘CT simulation.

## 6. Discussion

In Japan, 641,594 new cancer cases were diagnosed in 2003. According to the Research Group for Population-based Cancer Registration in Japan, the number of cancer cases in 2020 was projected to be 838,000 (501,000 males and 337,000 females) [11]. On the other hand, a structural survey by the Japanese Society for Therapeutic Radiation Oncology (JASTRO) has reported that the total numbers of new cancer patients and total cancer patients (new and repeat) treated with radiotherapy (RT) in 2005 were estimated at approximately 162,000 and 198,000, respectively, demonstrating an approximately 2-fold increase during the last decade [14]. The increasing number of cancer patients undergoing RT has resulted in a greater focus on estimating the requirements, including equipment, personnel, patient load, and geographic distribution, in order to identify and improve any deficiencies.

At GHMC, construction of the building and set-up of the treatment machine progressed on schedule. Major efforts were made to realize a compact prototype facility for C-ion RT based on the research and development of NIRS. The major benefit of downsizing the accelerator and beam delivery systems was cost reduction, and yet, its high performance was kept at the level of HIMAC. First, since the ratio of treatment frequency with the horizontal irradiation port (H-port) to that with the vertical one (V-port) was around 5:4, the facility needed three treatment rooms consisting of H-port, V-port, and H&V-port in order to treat more than 600 patients per year efficiently [15]. Second, the residual range of 250 mm covered the majority of patients [15]. The residual range depends not only on the beam energy, but also on the forming method of a lateral irradiation field and the irradiation-port length. Using the spiral-wobbler method, the energy of carbon-ions should be more than 400 MeV/n, corresponding to a 275-mm range, in order to obtain the 250-mm residual-range. Thus, the maximum energy was determined to be 400 MeV/n.

In clinical trials at NIRS, it was clear that carbon therapy was effective for respiratory moving targets such as lung and liver [5]. To treat these diseases, we adopted a relatively old technique of the beam delivery system, a beam wobbling method, range compensator or ridge filter for spreading out the Bragg peak. Using this technique for carbon therapy, we need to solve several problems in order to improve the accuracy of the treatments: (1) how to observe or measure the target movement that is an intra- or inter-fractional change of the target; (2) how to place the margin for the clinical target volume of the moving target; (3) how to design the range compensator in order to reduce the dose to organs at risk; (4) how, particularly with carbon therapy, to estimate the risk to normal tissues. These problems can obviously not be solved overnight, and in the meantime, techniques established at NIRS should be followed with scientifically accountable performance. In this respect, the accuracy of patient positioning will be analyzed during the daily treatments.

Recent technologic developments in the fields of accelerator engineering, treatment planning system, beam delivery, and tumor visualization have stimulated the process of transferring C-ion RT from physics laboratories to the clinic. Most of the clinical outcomes with C-ion RT alone have been published from Germany and Japanese facilities [4]. On the other hand, multimodality therapy such as concurrent chemotherapy and conventional radiotherapy has been standardized for locally advanced cancers in the head & neck, lung, esophagus, pancreas, bladder, and uterus *etc.* However, recurrence and increased toxicities are still problems to overcome. C-ion RT has the opportunity to demonstrate potential abilities in combination with surgery, cytotoxic drugs, molecular targeted drugs, and immunotherapy so as to increase local control, prevent severe toxicities, and maintain the quality of life. GHMC is the first C-ion RT facility in Japan to belong to a university hospital, as the two previous facilities NIRS and Hyogo Ion Beam Medical Center with C-ion RT in Japan consist of individual radiation oncology institutions. One of the advantages of our facility is its capability to perform multidisciplinary treatment for locally advanced tumors efficiently. In addition, expected or unexpected concomitant disease can be managed in collaboration with the appropriate department of the university hospital.

## 7. Conclusions

By using a compact prototype of the accelerator system, C-ion RT for the first patient at GHMC took place on schedule in March 2010. Based on the design and R&D studies carried out by NIRS, the size and cost of the machine could be reduced to one-third of those of NIRS. The facility set-up of GHMC was performed carefully regarding the facility design, treatment planning systems, and clinical preparations.

## Figures and Tables

**Table 1.** The basic specifications of the beam delivery system.

Treatment Rooms/Courses	3 Rooms and 4 Courses
	Room A: Horizontal
	Room B: Vertical + Horizontal
	Room C: Vertical
Residual Range	over 25 cm in water (400 MeV/n)
Irradiation Field Size	15 cm × 15 cm at maximum
	Beam Wobbling + Ridge Filter
Beam Broadening Method	Single Circle Wobbling Pattern
	Spiral Wobbling Pattern
SOBP Variations	2–14 cm in Water
Dose Rate	5 GyE/min (typical)
Irradiation Methods	Respiration-Gated Irradiation
	Layer Stacking Conformal Irradiation
